# Bridge Carbon Emissions and Driving Factors Based on a Life-Cycle Assessment Case Study: Cable-Stayed Bridge over Hun He River in Liaoning, China

**DOI:** 10.3390/ijerph17165953

**Published:** 2020-08-17

**Authors:** ZhiWu Zhou, Julián Alcalá, Víctor Yepes

**Affiliations:** Institute of Concrete Science and Technology (ICITECH), Universitat Politècnica de València, 46022 Valencia, Spain; zhizh2@doctor.upv.es (Z.Z.); jualgon@cst.upv.es (J.A.)

**Keywords:** greenhouse gas, environmental impact, cable-stayed bridge, life-cycle assessment, sustainable construction

## Abstract

Due to the rapid growth of the construction industry’s global environmental impact, especially the environmental impact contribution of bridge structures, it is necessary to study the detailed environmental impact of bridges at each stage of the full life cycle, which can provide optimal data support for sustainable development analysis. In this work, the environmental impact case of a three-tower cable-stayed bridge was analyzed through openLCA software, and more than 23,680 groups of data were analyzed using Markov chain and other research methods. It was concluded that the cable-stayed bridge contributed the most to the global warming potential value, which was mainly concentrated in the operation and maintenance phases. The conclusion shows that controlling the exhaust pollution of passing vehicles and improving the durability of building materials were the key to reducing carbon contribution and are also important directions for future research.

## 1. Introduction

With the rapid development of the world economy, infrastructure construction has made a giant leap. The total greenhouse gas emissions associated with the multiple phases of an infrastructure’s life cycle have accounted for 40% of global energy use [1]. According to the China Statistical Yearbook, it shows that in 2000, China consumed 56.929 million tons of oil in transportation, accounting for 24.9% of China’s total oil consumption. The Development Research Centre of the State Council of the People’s Republic of China forecasted that the country’s transport oil consumption would reach 256 million tons by 2020 [2]. Huge energy consumption leads to serious pollution of the natural and living environment, and meanwhile, the amount of greenhouse gases increases. Scientists and institutions around the world have proposed a series of measures and policies to alleviate the problems caused by the greenhouse gas effect [3,4].

Larsson Ivanov et al. [5] have investigated air pollution and greenhouse gas emissions from the production of certain building materials and products. They demonstrated that road transport is also a major source of greenhouse gas emissions. The Swedish Transport Authority has planned that the investment of infrastructure projects (such as bridges and tunnels) would increase by at least 5 billion Euros from 2020 to 2029, and that carbon dioxide emissions must be cut by between 17% to 30%.

In 2006, the Elinkaareltaan Tarkoituksenmukainen Silta Project was launched in Finland, Sweden, and Norway [6]. In 2009, Denmark joined in. The project aimed to optimize a bridge’s life cycle while covering economic, environmental, and aesthetic issues throughout the bridge’s life cycle, and they developed a life-cycle assessment (LCA) tool for bridges [7].

Using the LCA, the project developed openLCA, Efootprint, Ebalance, and other software. The key aim of the software system is to establish a strong database, including the Center for Environmental Assessment of Product and Material Systems ( SPINE@CPM) database of Sweden [8], Prozessorientierte Basisdaten (PROBAS) database of Germany [9], Environmental Management Association for Industry database of Japan (JEMAI) [10], The National Renewable Energy Laboratory of United States database (USNREL) [11], The Life Cycle Inventory of Universidad Real Instituto de Tecnologia de Melbourne(RMITLCI) database of Australia, the Swiss Ecoinvent database, and the European Reference Life Cycle Database (ELCD) have been established as complete databases [7].

The Ecoinvent database was created by several institutes using The Swiss Federal Institute of Technology Zurich domain name and the non-profit association Agroscope [12]. The database includes more than 2200 new data groups and 2500 updated data groups, which covers buildings, building materials, transportation, and so on. In addition to providing the summary data set, the database also includes the decomposed unit process data list, the data input and output of each production step, and the built data module. It provides a sufficient scientific research basis for LCA research in various fields.

In view of the increasing pollution of the environment by the construction industry, García-Segura et al. [13] and Itoh et al. [14] conducted carbon dioxide and cost assessments on box bridges. The study is a single example and lacks systematicity in the face of the construction of new types of bridges. Hong Wei [15] used life-cycle analysis and quantified the environmental impact of bridges. Heijungs et al. [16] presented research on framework modeling showing that there is insufficient practical guidance and proposed the establishment of a scientific framework for sustainable development life-cycle analysis in terms of products, materials, and technologies. Penadés-Plà et al. [17] used openLCA software to study the environmental impact of a box girder of two structural sizes, though the application reference value of actual engineering projects is insufficient. They studied the environmental impact contribution of box girder highway bridges under different maintenance schemes. In summary, the research results have laid the foundation for the research methods and ideas of the environmental impact of infrastructure. What is lacking is that the research is not comprehensive, systematic, and refined; the research and analysis are not comprehensive.

The comparison of case studies found that the combination of bridge structure design and aesthetics, human landscape and other concepts, the diversity of materials, the optimization of construction technology, rapid economic development, and the improvement of environmental requirements and other factors affect the bridge LCA, and a new assessment needs to be established. It is necessary to study the cause and effect process of “from the cradle to the end of life” at each stage of the entire life cycle. The comprehensive, meticulous, and rigorous research results that are in line with the bridge structure and bridge development form are more representative, important, and of higher quality data.

The above was the basis of the analysis of the thoughts and needs of this article. This study provides comprehensive research and analysis on the LCA of bridge structures and selected the comprehensive influencing factors of the four phases, from the cradle to the end of a completed three-tower cable-stayed bridge. In addition, the main causes and the mechanism of the environmental emission contribution in each stage were analyzed. Finally, the research results of the environmental emission contribution of cable-stayed bridges were obtained.

## 2. Methods

### 2.1. Research Framework and Method of LCA of a Cable-Stayed Bridge

This study used the openLCA 1.10.3 software [18], as well as the Ecoinvent database to study the contribution to the environmental impact of cable-stayed bridges. The LCA analysis of the cable-stayed bridge was divided into five phases: (1) cable-stayed bridge design, (2) cable-stayed bridge structural materials processing and construction, (3) cable-stayed bridge construction and installation, (4) cable-stayed bridge operation and maintenance, and (5) the decommissioning and dismantling of the cable-stayed bridge after the lifetime of the bridge. The reliability of the LCA results analysis mainly depended on the selection of a reasonable database and the accuracy of the parameters in each research stage. The study followed the ISO 14040:2006 framework [19] and the CML (Centrum voor Milieuweten schappen Leiden) 2001 standardized approach (Leiden University) [20]. According to the actual data of the whole process of cable-stayed bridges, the accuracy and effectiveness of the research and analysis were guaranteed. The verification information included the cable-stayed bridge design drawing, geological survey report, construction organization design, special plans, and the Ecoinvent database.

**Figure 1:**
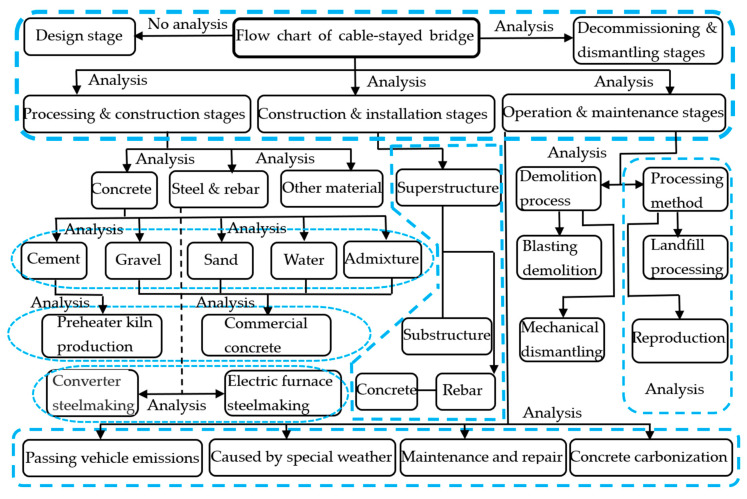
Life-cycle assessment (LCA) analysis flow chart of the three-tower cable-stayed bridge.

As shown in Figure 1, the LCA analysis of cable-stayed bridges was not carried out at the design phase. The bridge survey and design stage mainly consisted of the surveying and mapping of the engineering site by the design unit, as well as the interior design and production of the drawings. Large mechanical equipment and materials were not used at this stage, and only a small amount of prospecting and measuring equipment and the design work of building and structural engineers were required. As a whole, the environmental impact contribution of a cable-stayed bridge in this stage is not large; therefore, we did not analyze its effect.

### 2.2. Definition of the Environmental Impact Scope of a Cable-Stayed Bridge

Throughout its life cycle, the contribution of cable-stayed bridges to the environment is the impact of the entire process from the cradle to the grave. It is necessary to input all the data of each stage of the entire life cycle as input analysis data into the software and use part of the data generated in the process as output analysis data; for example, use concrete production data in the construction phase as input data and use waste concrete and wastewater generated during the production process as output data analysis.

International Standard (ISO, 2006b) provides an explanation [19]. The defining principle of the time dimension of the analysis mainly considers phases (1), (2), and (3), which should be implemented in accordance with the provision times of the design drawings. Stage (4) should be implemented in accordance with the design life for 100 years. Stage (5) should start calculating short-time emissions in accordance with the specification for 100 years from the beginning of the demolition to the completion of the landfill.

**Figure 2:**
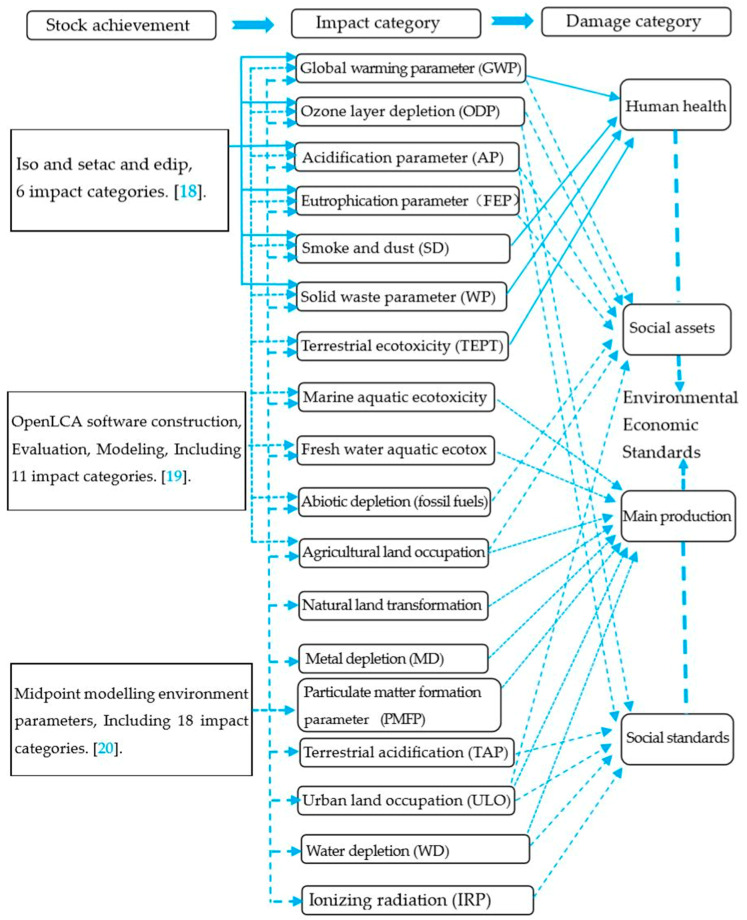
Schematic diagram of inventory and damage categories of construction materials’ life cycle list. ISO: International Organization for Standardization, SETAC: International Society for Environmental Toxicology and Chemistry, EDIP: Danish Industrial Product Environmental Design Method

In order to select the LCA impact assessment factor model framework of this article, the following three types of data were comprehensively studied: International Organization for Standardization (ISO), the International Society for Environmental Toxicology and Chemistry (SETAC), and the Danish Industrial Product Environmental Design Method (EDIP) to establish a framework (6 types), LCA software analysis factors (11 types), and midpoint modeling analysis (18 types), as shown in Figure 2 [17,19,21,22].

The main influencing factors and causes shown in Figure 2 show that the focus of building materials and whole-life research is on human health analysis and energy loss. Of the 18 types of influence parameters shown, this study focussed on the analysis of five major factors affecting the greenhouse effect, which according to Du et al. [23] and Kim et al. [24] are: global warming parameter (GWP), acidification parameter (AP), eutrophication parameter (FEP), particulate matter formation parameter (soot and dust PMFP), and solid waste parameter (WP).

### 2.3. Feature Modeling Method Selection and Weight Factor Analysis

In LCA modeling analysis, researchers mainly use two modeling methods: midpoint modeling and endpoint modeling [22,25]. In the full life-cycle analysis of the LCA process, the advantages and characteristics of the two methods should be comprehensively compared [26]. Each stage and each indicator adopts midpoint modeling, and the impact of bridge construction on human health and social assets adopts endpoint modeling. Penadés-Pla et al. [17] applied Kriging optimization and bridge modeling to find the midpoint and endpoint ranges.

Midpoint modeling typically involves selecting an indicator (the so-called midpoint) somewhere between the emissions and the endpoint in the environmental mechanism and modeling the impact of that indicator. The characteristic of midpoint modeling is that it does not pay attention to the overall environment mechanism, but the disadvantage is that there is uncertainty about the scope of the research, the duration of the research forecast, and the research model.

Endpoint modeling focusses on the representation of the contribution of LCA to the protected area. The representation model must include the entire environmental mechanism and attempt to model the process quantitatively. In the modeling process, the impact of modeling failure is not usually considered; thus, it is more uncertain and unknown. The potential benefit of this approach is that the effects at the endpoint level can be compared [12].

Considering the advantages and disadvantages of the two methods, the joint modeling and weighted analysis method of the midpoint and end-point were adopted in the modeling and analysis of a cable-stayed bridge [23] and parameter weighting was introduced into the LCA modeling process.

**Figure 3:**
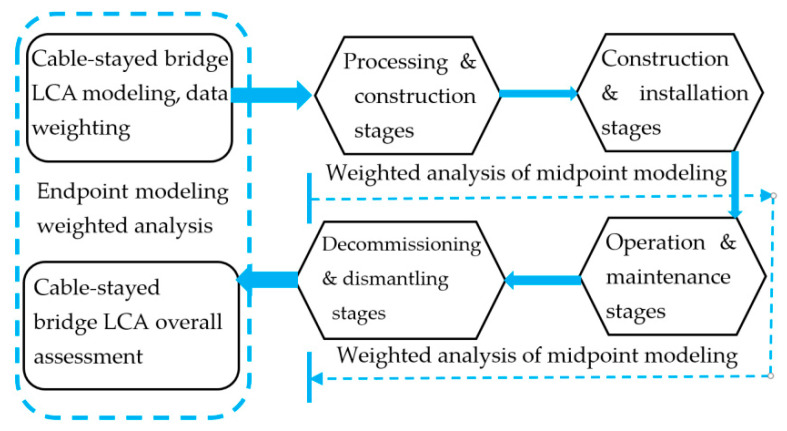
Schematic diagram of cable-stayed bridge modeling process.

**Figure 4:**
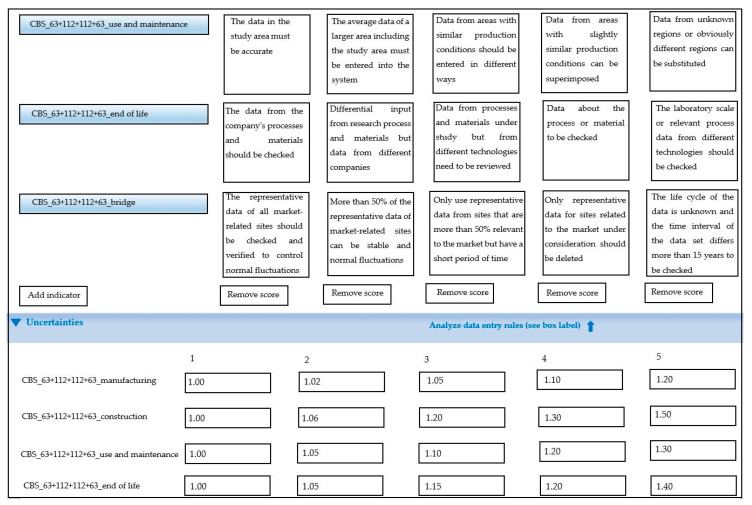
Schematic diagram of the LCA modeling process and weight coefficients.

As shown in Figure 3 and Figure 4, in the four phases of the cable-stayed bridge modeling and analysis process, the midpoint analysis modeling method was adopted, and the setting of weighted parameters was introduced. For the overall environmental impact assessment, the endpoint modeling research was adopted, the environment mechanism weighted parameters were introduced, and the feature modeling was introduced in the process of database selection and analysis process.

## 3. LCA Assessment Process and Data Analysis

This study selected the municipal bridge across the Hun He River in the Liaoning province of China as the research object. The bridge is a three-tower concrete cable-stayed bridge with a single cable plane. The bridge is 360 m long (63 + 112 + 112 + 63 + 10 m) with a 38 m surface width and a 2 m cable anchorage zone. The central bridge tower is a beam–tower–pier consolidating system and the bridge towers on both sides are a beam–tower consolidation system. A single-box double-chamber structure is adopted in the main beam with a 2.4 m central height, a 1% cross slope, a 25 cm top-plate thickness, a 24 cm bottom-plate thickness, a 2.5 m central web thickness, and a 1 m side web thickness. At every 6 m interval on the main beam, a transverse separating beam is set up with a 70 cm thickness and transverse beams are set up at the ends. The cable anchor is fixed at the bottom of the central web of a box-type beam. The main towers have a 20 m height over the bridge surface. An I-shaped cross-section (with a 5.0 m × 3.8 m dimension) was adopted for the upper tower columns and a solid cross-section (with a 5.0 m × 2 m dimension) was adopted for the intermediate tower columns. A box-type thin-wall structure was adopted for the pier body, a transverse separating plate was set up inside the pier, and the base is an extensive one. For the stayed cable, high-tensile galvanized steel wire, a chill-cast anchor, and a hot-extruded polyethylene (PE) guard sleeve were adopted. The main beams used in the bridge construction were precast hollow reinforced concrete slabs with a 65 cm thickness and a 125 cm width.

**Figure 5:**
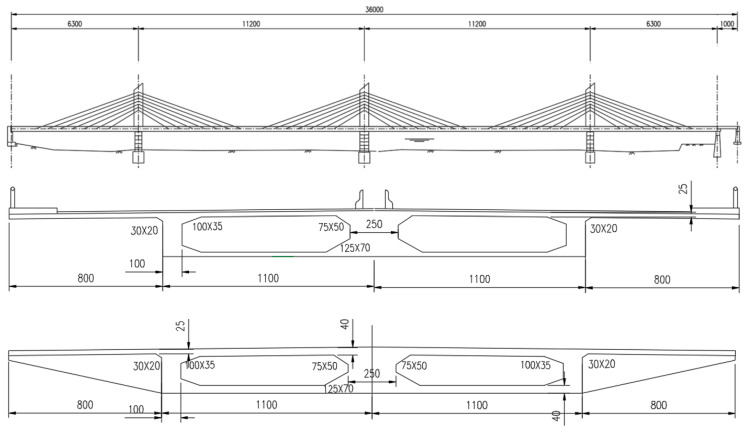
Three-tower cable-stayed bridge and the schematic diagram of the box girder structure.

As shown in Figure 5, the cable-stayed bridge is divided into three towers, four spans, and two cross-sections. The construction process was as follows: (1) First, adopting a cast-in-site caisson; second, hoisting to the pile position, digging, and discharging the soil in the well; third, sinking the pile to the designed bedrock position; and finally, pouring the slab concrete into the caisson. Continue to complete the pouring of the concrete of the dock. (2) The main beams were constructed with steel brackets with a “six + four” structure.

The construction was divided into three sections: assembling the outer mold of the steel formwork, assembling the inner mold of the box girder using a wooden mold, and inverting the outer mold and bracket three times. The inner mold of the box girder (damaged during the dismantling) was processed three times to finish the construction of the main girder. (3) The stay cable tension of the previous process was completed, and at the same time, the concrete of the subsequent process was poured, and constructed in order. (4) The last section of main beam concrete construction, bridge deck pavement, railing installation work was completed, and finally, the bracket was removed.

### 3.1. Processing and Construction Stage

The main materials of the cable-stayed bridge construction included concrete (comprising cement, water, gravel, river sand, and admixture), asphalt, steel bar, plate, rubber bearings, steel strand, steel products (steel plates, steel template, six-four military support), anchorage, corrugated pipe, wooden templates, wood, and other subsidiary materials.

#### 3.1.1. Concrete

The main ingredients of concrete are cement, water, gravel, river sand, and admixture. Cement is also the biggest contributor to environmental impacts. China’s cement production mainly adopts the new suspended preheater kiln production process, and the cement output produced by the new suspension preheating kiln production process accounts for 95% of China’s total annual cement output [27]. Each kind of building material has a physical and chemical environmental influence. According to the requirements of the construction drawings and the same-concrete data of the Ecoinvent database, ordinary Portland cement was selected. The database includes cement during the entire process, from the start to the finished product, including the source of upstream products (such as gypsum). The production of 1 kg of cement generates a waste heat emission of 0.135 MJ (standard deviation of 1.4918) [14]. There are four types of cable-stayed bridge concrete: C50, C40, C30, and asphalt concrete. The asphalt content is 6.5% and the density is 2.35 t/m^3^ [28]. The commercial concrete used in the cable-stayed bridge was supplied by local manufacturers. According to the database, the loss in the production process was determined to be 24.5 kg of waste per 1 m^3^ concrete. The sewage discharge value was 0.035 m^3^/m^3^. In the calculation of the environmental impact analysis, the concrete was divided into the production and construction phases, and the coefficient of the contribution of the emissions to the environmental impact is shown in Table 1.

#### 3.1.2. Main Material

Rebar, steel, and pipe were the main materials. Steel smelting in China is divided into two types [32,33]: converter steel and electric steel. A total of 90% of the steel output is made using converter steel and about 10% is made using electric steel [14]. The environmental impact contribution of a 1 kg steel bar discharge, which was determined using the database, is specified in Table 1.

#### 3.1.3. Material Transportation at the Manufacturing Stage

All the raw materials were ready to enter the site in the early phases of construction. According to the design plan, the transportation distance of concrete raw materials was 120 km, and the mixing water was tap water. The transportation distance of the commercial concrete was 30 km; the transportation distance of the steel bar, steel products, and steel strand was 160 km; the wood’s transportation distance was 80 km; and other materials were provided from the non-ferrous metal market, which was 100 km away. The materials were transported using three types of trucks: 17.5 m (49 t), 6.8 m (18 t), and 4.2 m (4 t). A gantry crane, a 25 t crane, and six erection workers completed the loading and unloading. The machines and tools are shown in Table 2.

The energy consumption value of the environmental impact contribution during the construction stage is given as:

(1) $$EC_{m}=\sum M_{qi}\times \lambda_{i},$$

where $EC_{m}$ is the value of environmental impact contribution of raw materials (kg), $M_{qi}$ is the mass of material *i* (kg), and $\lambda_{i}$ is the environmental emission coefficient of physicochemical material *i* (kg/kg).

The modeling calculation of the environmental impact of the transportation equipment is given as:

(2) $$T_{m}=\sum \left[(G_{qi}+G_{si})\times \left(\frac{D_{qi}}{100}\right)\times \left(M_{qi}/M_{ei}\right)\times \lambda_{n}\right]$$

where $T_{m}$ is environmental impact contribution of the transport equipment (kg), $G_{qi}$ is the fuel consumption of the truck load (L/100 km), $D_{qi}$ is the freight car transport distance (km), $G_{si}$ is the non-load fuel consumption of freight cars (L/100 km), $M_{qi}$ is the total mas of the material, $M_{ei}$ is the load capacity per vehicle (kg), and $\lambda_{n}$ is the physicochemical environmental emission coefficient of of oil *n* (kg/kg).

The construction of the environmental impact model of loading and unloading machinery and personnel is given as:

(3) $$M_{m}=\sum (T_{qi}\times K_{mi}\times \lambda_{n})+\sum \left(P_{mi}\times \lambda_{p}\right),$$

where $M_{m}$ is the environmental impact contribution of the loading and unloading machinery and personnel (kg), $T_{qi}$ is the consumption per mechanical equipment (L/shift), $K_{mi}$ is the total number of shifts (working days), $P_{mi}$ is the number of stevedore shifts (working days), and $\lambda_{p}$ is the numerical coefficient of environmental impact contribution of personnel per working day (kg/working day).

The total environmental impact contribution at the construction stage is given as:

(4) $$C_{m}=EC_{m}+T_{m}+M_{m}.$$

### 3.2. Construction and Installation Phases

The construction and installation phases were the main phases of the cable-stayed bridge’s environmental impact contribution to the research. The construction of various products required the joint work of a large quantity of mechanical equipment and construction personnel. The environmental impact mainly included the following parts.

#### 3.2.1. Environmental Impact Contribution of Materials Processing

Due to the need for reinforcement, steel, steel wire, and other raw materials were sent to the site. The technicians performed the processing, lashing, and installation of the steel bars according to the construction design drawings. See Table 3 for the machinery and equipment used in the construction process.

#### 3.2.2. Environmental Impact Contribution of the Machinery and Equipment in the Processing and Construction Stage

The calculation of the environmental impact model of mechanical equipment is given as:

(5) $$C_{m}=\sum \left(E_{mj}\times T_{j}\times \lambda_{j}\right),$$

where $C_{m}$ is the environmental impact contribution value of mechanical equipment (kg), $E_{mj}$ is amount of fuel or power consumption of equipment *j* (L/hour, kW/hour), $T_{j}$ is the total working hours of equipment *j*, and $\lambda_{j}$ is the fuel or electricity physicochemical environmental emission coefficient of equipment *j* (kg/l, kg/kW).

#### 3.2.3. Value of the Environmental Impact Contribution of Managers and Skilled Workers

The environmental impact modeling calculations for managers and skilled workers is done using:

(6) $$P_{m}=W_{m}\times \lambda_{p}\times T_{p},$$

where $P_{m}$ is the value of the environmental impact contribution of skilled workers (kg), $W_{m}$ is the total number of workers (people), $\lambda_{p}$ is the environmental impact coefficient of workers (kg/day/worker), and $T_{p}$ is the total time worked (days).

According to the construction organization design, there were 36 project management and technical staff, 180 technical workers on average, 24 logistics service staff, and the construction period was 14 months.

#### 3.2.4. Contribution Value of the Electric Power Energy to the environment during the Construction Period

The calculation of the power energy environmental impact modeling during the construction period is given as:

(7) $$E_{m}=\sum \left[T_{i}\times \lambda_{i}\times \left(1+L_{m}\right)\right]+G_{m}\times \lambda_{n}\times T_{m}\times \left(1+L_{n}\right),$$

where $E_{m}$ is the environmental impact contribution value of the electricity and oil consumption during construction (kg), $T_{i}$ is the power consumption (kWh/day) of personnel (managers, skilled workers, etc.), $\lambda_{i}$ is the physical-chemical environmental emission coefficient of the power consumption (kg/degree), $L_{m}$ is the power loss value (degree/day), $G_{m}$ is the amount of oil consumed by the generator in a power outage and during field operations (kg/hour), $\lambda_{n}$ is the physicochemical environmental emission coefficient of oil class *n*, $T_{m}$ is the total working time (hours) of the equipment, and $L_{n}$ is the oil loss during the generator operations (kg/hour).

#### 3.2.5. Values of the Contribution of Project Managers and Technical Workers to the Garbage and Sewage Environment

The calculation formula of the environmental impact model of waste and pollutants generated by managers and skilled workers is given as:

(8) $$M_{p}=P_{a}\times T_{m}\times T_{n}\times \lambda_{p}+S_{m}\times P_{a}\times \lambda_{x}\times T_{n},$$

where $M_{p}$ is the contribution value of the garbage and sewage environmental impact from staff during the life of the project (kg), $P_{a}$ is the total number of project personnel (people), $T_{m}$ is the quantity of household garbage (kg/day), $T_{n}$ is the working time of the staff on duty (days), $\lambda_{p}$ is the environmental emission coefficient of the household garbage (kg/kg), $S_{m}$ is the discharge quantity of personnel (kg/day), and $\lambda_{x}$ is the environmental emission coefficient of the pollutant discharge (kg/kg).

The total environmental impact contribution during the construction stage is given as:

(9) $$B_{m}=C_{m}+P_{m}+E_{m}+M_{p}.$$

**Figure 6:**
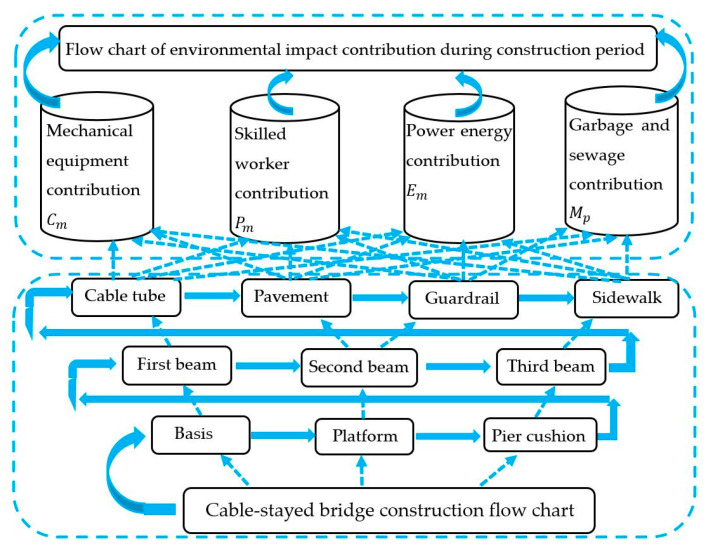
Schematic diagram of the process flow and environmental impact contribution during the construction.

The construction of the three-tower cable-stayed bridge was completed according to the flowchart shown in Figure 6, which saved materials, increased the working time of the mechanical equipment and the number of skilled workers, and improved the environmental impact contribution.

### 3.3. Operation and Maintenance Stage

After the completion and acceptance of the cable-stayed bridge, it entered the operation and maintenance stage. The cable-stayed bridge is an integral part of the municipal road. After the cable-stayed bridge was completed and put into use, the local municipal road maintenance department was responsible for the daily maintenance and repairs of various types of damage. An analysis of the statistical data published by the maintenance department shows that the main content of the maintenance work is divided into daily maintenance for more than five years, monthly maintenance and inspection, annual maintenance, revision, and replacement [34]. After the beginning of the operation phase, a large number of motorized and non-motor vehicles pass every day; therefore, it was necessary to analyze the environmental pollution values of exhaust emissions [35].

#### 3.3.1. The Amount of Environmental Impact Contribution for Maintenance of the Cable-Stayed Bridge

Table 4 summarises the period and content of the maintenance of the cable-stayed bridge. The data analysis was calculated according to the content of the table. The value of the contribution of the maintenance and maintenance environment caused by the impact of the natural environment in the table is uncertain. The analysis of attendance and the calculation of maintenance workers are also added.

The environmental impact contribution of the cable-stayed bridge maintenance:

(10) Cm=∑[Bm×(TmTp)×λp]+∑[Pm×(TmTn)×λn]+Cv,

where $C_{m}$ is the value of the environmental impact contribution to maintenance and repair (kg), $B_{m}$ is the bridge deck pavement replacement area (m^2^), $T_{m}$ is the service life of the bridge design (years), $T_{p}$ is each replacement time of the bridge deck pavement (years), $\lambda_{p}$ is the environmental emission coefficient of the bridge deck pavement (kg/kg), $P_{m}$ is the coated area of the pier column (m^2^), $T_{n}$ is the pier painting change time per time (years), $\lambda_{p}$ is the environmental emission coefficient of the pier coating (kg/kg), and $C_{v}$ is the environmental impact contribution value of the mechanical equipment during the maintenance stage (kg).

#### 3.3.2. Environmental Impact Contribution of Vehicles during the Operation of the Cable-Stayed Bridge

Transportation accounts for 26% of the global energy consumption and 23% of greenhouse gas emissions are energy-related. Street traffic accounts for 74% of the world’s transport sector traffic [36].

The cable-stayed bridge is part of a municipal road, which is used by a large number of vehicles every day and is a major contributor to global greenhouse gases. Colvile et al. [31] have shown that diesel, gasoline vehicle exhaust, liquid gasoline, and gasoline evaporation account for at least 50% of volatile organic compounds (VOC) in the environment. The chemical substance balance (CMB) and the proportion in the emissions inventory [39], which is determined using the chemical substance balance (CMB), are much greater than the proportion of paint and solvent contributions [40].

To obtain detailed data about the relevant traffic on the cable-stayed bridges, data can be searched for in the traffic database [41]. The total length of roads in Fushun in 2019 was 6911.4 kilometers, with an annual highway freight turnover of 1277.295 million tons and highway passenger turnover of 1052.48 million kilometers, with 262,000 civilian cars and 19,035 trucks [33]. According to statistical research results, carbon emissions from passenger cars in China are estimated to be 305.4 g/km, and carbon emissions from trucks are estimated to be 271.8 g/km [39].

The calculation of the environmental impact model during operation is given as:

(11) $$T_{m}=\sum_{1}^{100}\left[C_{m}\times K_{m}\times \lambda_{c}\times V_{m}\times K_{m}\times \lambda_{t}\right]\left(1\pm \lambda_{y}\right),$$

where $T_{m}$ is the environmental impact contribution during operation (kg); $C_{m}$ and $V_{m}$ are the annual toll of passenger cars and trucks (set), respectively; $K_{m}$ is the passenger car journey distance on the cable-stayed bridge (km); $\lambda_{c}$ and $\lambda_{t}$ are the environmental emission coefficients of passenger cars and trucks (kg/kg), respectively; $\lambda_{y}$ is the annual increase or decrease of passenger cars and freight cars (%); and 100 is design life (years).

#### 3.3.3. External Environmental Impact Contribution Value of the Cable-Stayed Bridge

During the operational period, the environmental impact contribution of the cable-stayed bridge under the influence of special weather, such as snow, rain, and dust, was analyzed by referring to the monitoring data of the local environmental protection department. Monitoring data from 2010 to 2019 show that: GWP = 1.3~2.2 mg/m^3^, AP = 12~39 mg/m^3^, FEP = 22~39 mg/m^3^, PMFP = 29~46 mg/m^3^, and WP = 50~78 mg/m^3^ [35].

The environmental impact contribution of the cable-stayed bridge should be determined according to the monitoring data. Considering the declining trend of the values of the five indices year by year, it was found from the statistical data over 10 years that the values kept changing by about 30%, and that the changes of the values decreased in the later period under a favorable environment. The influence of the changed values was not considered in this study.

#### 3.3.4. The Value of the Contribution of Concrete Carbonation to the Environmental Impact of the Cable-Stayed Bridge

Concrete carbonation was mainly affected by its performance and external environmental factors. The temperature, carbon dioxide (CO_2_) concentration, and relative humidity greatly influence carbonation, which also determines the carbonation depth and compressive strength of concrete [41]. Chen et al. [36] and some other studies have established a multi-field coupling numerical model and action quantization index for the carbonation analysis of Martínez-Muñoz et al. [42], and have quantitatively analyzed the influence of temperature, relative humidity, CO_2_ concentration, and other factors on the concrete carbonation depth by introducing an environmental correction coefficient.

The model relation of the quantity of CO_2_ absorbed by per unit volume of concrete is determined using:

(12) $$m_{0}=\left(1-\alpha \right)\times 8.22B$$

where $m_{0}$ is amount of CO_2_ absorbed by ordinary Portland cement concrete (mol/m^3^), *B* is the amount of cement material per unit volume of concrete (kg/m^3^), and *α* is the content of mixed materials in ordinary Portland cement (%).

The calculation of the numerical model of the total carbonation of concrete is done using:

(13) $$K_{m}=N_{c50}m_{50}+N_{c40}m_{40}+N_{c30}m_{30},$$

where $K_{m}$ is total carbonation amount of concrete (kg); $N_{c50}$, $N_{c40}$, and $N_{c30}$ are the volumes of C50, C40, C30 concrete (m^3^), respectively; and $m_{50}$, $m_{40}$, and $m_{30}$ are the carbonation moduli of C50, C40, and C30 concrete (kg/m^3^), respectively.

The environmental impact contribution value during the operation and maintenance of a cable-stayed bridge (external environmental impact quantity in kg) is given as:

(14) $$M_{m}=C_{m}+T_{m}+S_{m}+K_{m}.$$

### 3.4. Abandonment and Demolition Stage

The designed service life of highway bridges in China is 100 years. The service life of bridges is shortened under the condition of long-term exposure to the $Cl^{-}$ or CO_2_ harsh environments. After several bouts of maintenance, the designed service life of bridges is determined to enable the designed service life to be reached, before being abandoned and dismantled [38].

There are two commonly used demolition schemes: manual demolition with mechanical equipment and blasting demolition. The safety factor of demolition via blasting the cable-stayed bridge, which is located in the urban area, is low. Through a comprehensive evaluation, the plan of mechanical and manual demolition was adopted. The comprehensive plan of segmental cutting, segmental hoisting, site crushing, and freight car transportation to the pre-burial site and steel mill was determined from the aspects of technology, safety, economy, etc.

Referring to the demolition experience of similar bridges, the mechanical equipment requires 6 long-arm crushers, 4 loaders, 10 heavy-duty transport vehicles, 4 steel transport vehicles, and 30 management and technical workers. Demolition is scheduled to take three months. The generated environmental impact contribution coefficient was calculated by referring to Table 2. The crushed concrete waste is to be transported and buried in a landfill, which is 160 km away. The steel scrap is to be transported to a steel mill, 180 km away, for smelting. According to the study results of Kim et al. [43], and in combination with the bridge removal scheme, it was determined that the recovery rate of concrete is 95%, the recovery rate of steel is 72%, and the recovery rate of steel and steel strand is 85%.

## 4. Results and Discussion

### 4.1. Summary and Analysis of the Environmental Impact Contributions at Each Stage

The LCA analysis process of a cable-stayed bridge was completed, the data were summarized, and the impact of each stage on the environmental impact contribution was analyzed. Table 5 shows the statistics of the main engineering materials and auxiliary engineering materials project of the three-tower cable-stayed bridge.

Since the cross-sectional structure of the cable-stayed bridge was divided into two sections, for the analysis and calculations, 1 m^2^ was selected as the LCA research unit, and the mix ratio of C50, C40, and C30 concrete was selected according to the ratio provided by the Ecoinvent database.

The statistical data of the main engineering materials and auxiliary engineering materials of the three-tower cable-stayed bridge are shown

**Figure 7:**
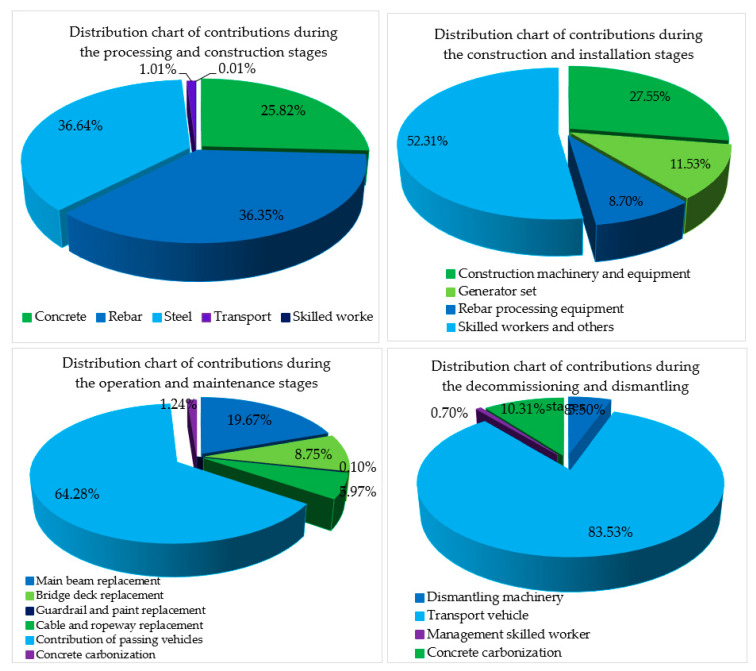
Schematic diagram of the environmental impact contribution of each stage of the cable-stayed bridge.

As shown in Figure 7, the environmental impact contribution value of the cable-stayed bridge in each stage, and the environmental impact contribution value of the steel products and steel bar in the processing and construction phases, accounted for 36.64% and 36.35% of the total amount, respectively. The main reason for this result is that China’s steelmaking process is mainly concentrated in the converter steelmaking process. Steel has a great impact on the environment during the production process, according to the study results of Zhu et al. [44]. Therefore, it is necessary to improve the steelmaking process and technical level and pay more attention to the development and application of low-carbon environmental protection technologies.

In the construction and installation phases, the environmental impact contribution was mainly due to skilled workers and the energy consumed by project participants (electricity, drinking water, accommodation materials), along with the wastewater dumped by the project participants for cooking and washing, which accounted for 52.31%. The cable-stayed bridge was a municipal project, which needed a large number of management technicians. All of these people who lived on the construction site for a long time were responsible for the large number of contributions to environmental impact.

The main reason for the increase in the value was that the environmental impact contribution of the materials was reduced but the environmental impact contribution of personnel and mechanical equipment was increased. The environmental impact contribution of the mechanical equipment reached 27.55% of the total amount.

In the operation and maintenance stage, the environmental impact contribution caused by vehicle traffic was dominant, accounting for 64.28% of the total, which is a number that needs to be taken seriously by the automotive and transportation sectors. The environmental impact contribution of vehicles can no longer be underestimated; although the designers and researchers are looking for ways to reduce the environmental impact contribution at other phases, there is still little they can do about it.

The value of the environmental impact contribution in the abandonment and demolition stage was mainly caused by concrete waste, steel, and steel waste removed by vehicle transportation, which accounted for 83.53% of the total amount.

The cable-stayed bridge will have a significant impact on the environment after 100 years of operation. This can be seen in the numerical results in Figure 6, which show that vehicle traffic was the main cause of environmental pollution and it also affected the environmental impact contribution of the entire cable-stayed bridge.

### 4.2. Summary and Analysis of the Environmental Impact Contributions of Five Indicators of the Cable-Stayed Bridge

**Figure 8:**
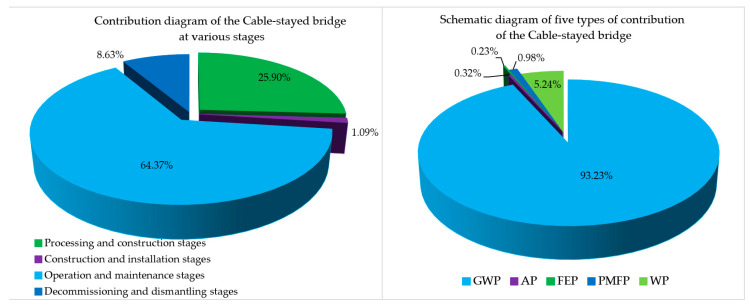
Schematic diagram of the environmental impact contribution of the four phases of the cable-stayed bridge.

As shown in Table 6, the total environmental impact contribution of GWP, AP, FEP, PMEP, and WP in the four phases of the three-tower cable-stayed bridge was 19,3013,584.7 t. As shown in Figure 8, the processing and construction phases accounted for 25.90%, the construction and installation phases accounted for 1.09%, the operation and maintenance phases accounted for 64.37%, and the abandonment and demolition phases accounted for 8.63%. The main reason for the huge impact on the environment during the operation and maintenance phases was that the replacement and maintenance of cable-stayed bridge structural components during its 100-year design life will affect the environment. According to the service life of its components (Table 4), the pre-stressed steel strands of the stay cables installed on the cable-stayed bridge need to be replaced three times (within 100 years of service life). The bridge deck pavement will be replaced 10 times. The drainpipe of the bridge will be replaced twice. The bridge anti-collision railing will be replaced twice. The exposed concrete waterproof layer of the bridge will be replaced 10 times.

Among the environmental impact values for the cable-stayed bridge, the carbon dioxide (CO_2_) emissions accounted for 93.23% of the total emissions in Figure 7, which was one of the reasons for choosing the five types of research parameters. The other 13 types of environmental impact values were relatively small. It can be seen that, in the future, global warming due to gas emissions and its precise and detailed research and analysis should be the focus of researchers in the construction industry.

This is due to the following three reasons: the large amount of transport waste, the large number of used transport vehicles, and the long transport distance.

**Figure 9:**
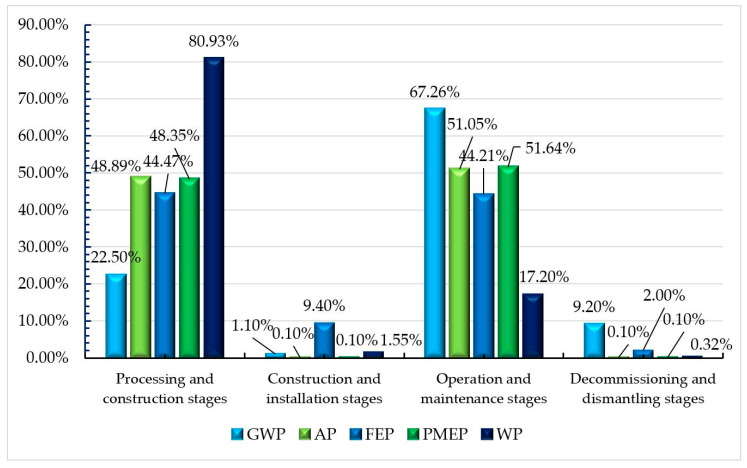
Schematic diagram of the environmental impact contribution of the four-stage cable-stayed bridge.

As shown in Figure 9, the environmental impact contribution of the cable-stayed bridge mainly focussed on the processing and construction phases and the operation and maintenance phases. The environmental impact contribution of these two phases was mainly concentrated on the production of steel bars and steel products and the contribution of the exhausts from the passing vehicles, accounting for 73% and 64.28% of the environmental impact contribution of each stage, respectively. How to better reduce the environmental impact contribution in the future is worthy of in-depth consideration by researchers, designers, and managers. The overall LCA environment contribution order of the cable-stayed bridge was: GWP (93.23%) > WP (5.24%) > PMFP (0.98%) > AP (0.32%) > FEP (0.23%). The highest proportions of GWP, PMFP, and AP in the operation and maintenance phases were 67.26%, 51.64%, and 51.05%. The highest proportions of WP and FEP in the processing and construction phases were 80.93% and 44.47%.

**Figure 10:**
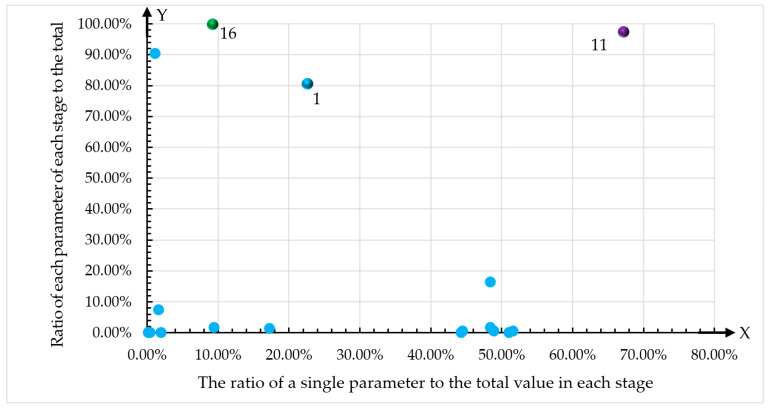
Distribution map of the points of five environmental impact contributions of the cable-stayed bridge.

As shown in Figure 10, the environmental impact contribution of point 11 was the largest. The environmental impact contribution of point 1 was the second-largest, and the environmental contribution impact of point 16 was the third-largest; The environmental impact contributions of the other points were much lower.

**Figure 11:**
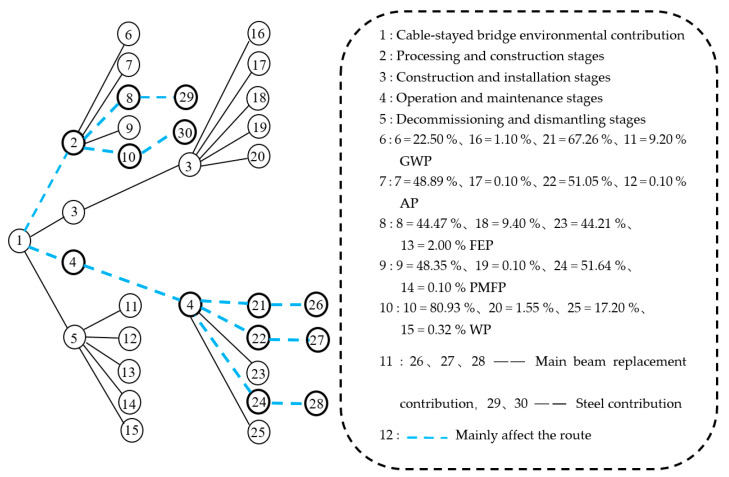
Schematic diagram of the Markov chain probabilities of the cable-stayed bridge.

For Figure 11, the percentages show that the GWP in the operation and maintenance stage accounted for 67.26% of the total GWP of the cable-stayed bridge, and accounted for 97.40% of the total environmental contribution during the operation and maintenance phases. For point 1, the percentages show that the GWP in the processing and construction phase accounted for 22.50% of the total GWP of the cable-stayed bridge and 80.80% of the total environmental contribution in the construction and installation phases. Finally, the environmental impact of point 16 was much lower, and percentages show that the demolition phase GWP accounts for 9.20% of the total GWP of the cable-stayed bridge, and the abandonment and demolition phase GWP accounted for 99.70% of the total environmental contribution in the abandon and demolition phase.

Through the research and analysis in Section 4.1 and Section 4.2, it was concluded that the environmental influence factors of cable-stayed bridges were divided into five index levels, and the influence factors of each index level had an impact on the environment.

The study proposed a Markov chain model capable of considering the maintenance factors used by Li et al. [45]. The Markov chain model can solve the problem of multiple factors. The given Markov chain probability diagram shows the ratio of the environmental impact factors at each stage of the cable-stayed bridge (Figure 11). The data comes from Table 5.

As shown in Figure 10, the environmental impact contributions of the cable-stayed bridge were mainly from the processing and construction phases, which manifested as FEP = 193,738.3 kg and WP = 8191,263.9 kg, respectively. During the operation and maintenance phases, the contributions were GWP = 121,031,298 kg, AP = 317,034.6 kg, and PMEP = 979,739.5 kg, respectively.

## 5. Conclusions

This study analyzed a three-tower cable-stayed bridge in China. First, this research studied and analyzed the definition of environmental impact assessment parameters, omitted the set of 13 parameters with small impact, and focussed on the analysis of the five parameters by applying midpoint modeling. The final endpoint modeling analysis conclusion verified the accuracy and effectiveness of this method. The amount of CO_2_ emission in the environmental impact value GWP of the cable-stayed bridge accounted for 93.23% of the total emissions.

Second, the analysis data of the cable-stayed bridge adopted the data analysis of the whole bridge. The bottom and topside parts of the bridge were all taken as the analysis object. The environmental impact contribution of the concrete production stage was classified into the production and construction phases for analysis because the cable-stayed bridge is a municipal project and it uses commercial concrete. Therefore, the environmental impact contribution of the construction and installation phases was mainly influenced by management technicians, accounting for 52.3% of the emissions during this stage. At the same time, it shows that in the process of the LCA analysis, data classification analysis should be set according to the actual situation of the project, which makes the results more scientific and practical.

Third, the operation and maintenance stage of the cable-stayed bridge was the main aspect that contributed to the environmental emissions, since most of the structural components of the cable-stayed bridge were replaced 2 to 10 times during their lifetime, and each change had a significant impact on the environmental impact contribution. Combined with vehicle exhaust emissions, this resulted in a 64.37% environmental impact contribution of the operations and maintenance phases, where the specific environmental impact contribution value was 124,307.2 t.

Finally, each stage contributed to the environmental emissions, and the numerical value reflected the degree of environmental impact. In the last two phases in particular, carbonation had a greater impact on the environmental impact contribution of the last stage. Especially in the operation and maintenance phases, concrete carbonation absorbed 1712.9 tons of CO_2_, which made an important contribution to the environmental impact. At the same time, the carbonation of the concrete opened up corrosion channels for the steel bars of the structural components, resulting in maintenance and replacement during the operation stage, and finally, the cable-stayed bridge was demolished.

There are still some defects in this study. These lie in the insufficient analysis of the data of equipment loss and damage caused by daily accidents, and environmental impact assessment caused by the natural environment, such as earthquakes, tsunamis, and hurricanes. The data analysis, theories, and methods of modeling used in this study can be used as a reference for research in this field. Furthermore, the research results can provide ideas and references for researchers and managers to study the whole-life analysis of a basic bridge.

## Figures and Tables

**Table 1:** Environmental impact contribution coefficient of materials during the processing and construction [29,30,31].

Material Name	Unit	GWP	AP	FEP	PMFP	WP
P. I. 52. 5	kg/t	1042.00	0.28	1.61	2.24	0.00
P. O. 42. 5		920.00	0.25	1.43	2.02	0.00
P. S. 32. 5		678.00	0.20	1.09	1.57	0.00
River sand	kg/m^3^	2.56	0.00	0.00	0.00	0.00
Gravel	kg/m^3^	3.30	0.00	0.00	0.00	0.00
Flake	kg/m^3^	3.37	0.00	0.00	0.00	0.00
C50	kg/m^3^	705.00	0.02	0.02	0.05	0.00
C40		608.00	0.01	0.01	0.05	0.00
C30		565.00	0.01	0.01	0.04	0.00
Ordinary asphalt	kg/t	174.00	0.00	0.00	0.17	0.11
Modified asphalt		296.00	0.00	0.00	0.17	0.11
Grade I and II steel bars	kg/t	4524.00	46.10	28.60	158.40	258.00
Steel wire		3551.00	46.10	28.60	158.40	258.00
Large steel	kg/t	4339.00	56.60	34.80	150.10	323.00
Medium steel		3589.00	46.60	28.90	124.60	268.00
Small steel		3560.00	46.10	28.60	123.40	252.00
Diesel	kg/kg	4.62	0.00	0.00	0.00	0.00
Gasoline	kg/kg	4.36	0.00	0.00	0.00	0.00
Waterproof coating	kg/kg	0.41	0.16	0.02	0.00	0.02
Power consumption	kg/kwh	0.98	0.00	0.00	0.00	0.00
Consumption	kg/person/day	2.88	0.00	0.13	0.00	0.50

GWP: Global warming parameter; AP: Acidification parameter; FEP: Eutrophication parameter; PMFP: Particulate matter formation parameter; WP: Solid waste parameter.

**Table 2:** The statistical table of used machinery and equipment at each stage [32,34].

Vehicle, Machinery Type	Fuel Consumption (kg/km)	Transport Distance (km)	Types of Shipping Materials
Heavy truck (49 t dead weight, length 13–17.5m)	0.67–0.84	120, 160	Cement-crushed stone-river sand; steel bar, steel strand, and other steel products
Medium-sized truck (18 t dead weight, length 5.8–6.8 m)	0.15–0.21	120, 80	Additives, wood
Light truck (4 t dead weight, length 2.6–4.2m)	0.10–0.14		
Concrete mixer truck (12 m^3^)	0.13–0.16	30	C50, C40, C30
Gantry crane (50 t)	22–30 kW	0.5	Lifting steel bar, steel strand, and other steel products
Crane (25 t)	0.13–0.18	2	Lifting Rebar, steel strand, and other steel products

Remarks: 0 # Diesel = 0.835 kg/L, +10 # Diesel = 0.85 kg/L, −10 # Diesel = 0.84 kg/L. The truck used 0 # Diesel.

**Table 3:** Summary table of the operation data of medium and small machines and equipment during construction and installation.

Device Name	Specification Model	Quantity (Table)	Power (KW)	Use Time (Month)	Oil (L/hour)
Excavator	PC400-1	1	228	6	35~45
Roller	YZ20Ton	1	249	2	25~35
Loader	ZL50	2	162	6	14~15
Dump Truck	CQ33 (L/100km)	2	380	2	50~60
Sprinkler	11.7m^3^ (L/100km)	1	22.5	10	15
Engineering rig	XR360 Rotary drilling rig	2	298	1	20~30
Engineering rig	Impact drill JK-6	1	200	1	10~15
Concrete pump truck	SY5125THB-9018III	1	176	1	0.5~0.6
Concrete transport truck	12m^3^ (L/100km)	6	240	6	8~10
Car crane	QT25	2	213	3	4~6
Mortar mixer	HZS180	2	120	2	120 kw
High frequency vibrator	ZG50	30	2	6	2 kw
Diesel generator sets	500KW	1	500	2	131
Rebar cutting machine	GT5-12	1	94	2	94 kw
Steel bending machine	GW 40	2	40	2	40 kw
Rebar cutting machine	GQ50	2	50	2	50 kw
Profile cutting machine	J3G-AL-400	1	400	1	400 kw

Remarks: The normal operation of the concrete pump truck is 40 cubic meters per hour.

**Table 4:** Summary table of the cable-stayed bridge maintenance, along with its maintenance cycle and causes [36,37,38].

Bridge Disease	Causes	Conservation Measures	Maintenance and Repair Cycle
Concrete carbonation, disease	Shock, vibration, overload, uneven settlement, chemical erosion, abrasion, blowing, freezing and thawing	Brush protective layer, repair cracks, recast pavement	The main beam is replaced every 50 years, the bridge deck pavement and the waterproof layer are replaced every 10 years, the main beam body is maintained every 5 years, and the common repair is every 2 years
Rebar disease	Chloride corrosion, concrete carbonation	Eliminate leakage points, crack closure, repair of protective layer, zinc coating protection	Consider carbonized corrosion, repair once every 70 years, the pre-stressed steel strands of the stay cables are replaced every 20 years
Component performance degradation and maintenance	Overload, aging	Local repair, component replacement	Piers and bearing caps are painted every 5 years, rubber bearings are replaced every 25 years, and expansion joints are replaced every 10 years
Probability of failure	External damage, exceeding design life	Repair, replacement	Deck drainage pipes are replaced every 50 years (the repair is every 2 years), anti-collision guardrails are replaced every 15 years (the repair is every 5 years), lighting devices are replaced every 50 years (the repair is every 5 years)
Unpredictable external environmental impact	Car accident, overload, collision, bad weather	Repair, replacement	Repair and replace at any time

**Table 5:** List of environmental impact criteria for a cable-stayed bridge at each stage.

Environmental Parameters	Unit	Processing and Construction Phases	Construction and Installation Phases	Operation and Maintenance Phases	Decommissioning and Dismanting Phases
GWP	kg	40,425,577.87	1,906,820.13	121,031,298.3	16,574,524.2
AP	kg	303,615.4	14.4	317,034.68	275.44
FEP	kg	193,738.31	40,772.73	192,615.69	8574.12
PMFP	kg	917,232.2	6.6169	979,739.53	187.68
WP	kg	8,191,263.94	156,810.04	1,740,820.94	32,862.67

**Table 6:** Statistical table of raw materials and accessory materials of the cable-stayed bridge.

Material Name	Unit	Quantity	Number	Material Name	Unit	C50	C40	C30
C50 concrete	m^3^	9050	15	Cement	m^3^	1337	213	560
C40 concrete		1761	16	Fly ash		315	54	124
C30 concrete		4714	17	Gravel		3693	770	1687
Asphalt concrete		447	18	Sand content		3102	629	2082
Rebar	Level I (Ton)	2037.5	19	Water		602	95	262
	Level II (Ton)	37	20	Steel	Ton	191.6		
Plate rubber support	m^3^	2.33	21	Bellows	ø127 (m)	3679		
Stranded wire	ø15.24 (Ton)	425.8	22		ø90 (m)	7783		
Lasso	Ton	350.3	23		ø80 (m)	3670		
Cable anchor	Set	168	24		90 × 19 (m)	28,428		
Anchor	15——27 (Set)	240	25	Military beam, steel pipe bracket	Ton	2234		
	15——14 (Set)	126	26	Box beam steel formwork	Ton	384.1		
	15——9 (Set)	176	27	Box beam inner model	m^2^	21,600		
	15——5 (Set)	1496	28	Fang Mu	m	9600		
